# Editorial: Human brain banking – Bridging brain health and precision neurology

**DOI:** 10.3389/fneur.2023.1322200

**Published:** 2023-10-27

**Authors:** Yue Huang, Qiao-Xin Li, Ling-Xiao Cao, Gang Wang, Daniel Kam Yin Chan, Conceicao Bettencourt, Adrienne E. Milward

**Affiliations:** ^1^Human Brain and Tissue Bank, China National Clinical Research Center for Neurological Diseases, Beijing Tiantan Hospital, Capital Medical University, Beijing, China; ^2^Department of Neurology, Beijing Tiantan Hospital, Capital Medical University, Beijing, China; ^3^Department of Pharmacology, Faculty of Medicine and Health, School of Biomedical Sciences, UNSW Sydney, Sydney, NSW, Australia; ^4^National Dementia Diagnostics Laboratory, The Florey Institute, University of Melbourne, Parkville, VIC, Australia; ^5^School of Medicine, Ruijin Hospital, Shanghai Jiao Tong University, Shanghai, China; ^6^Department of Aged Care and Rehabilitation, Bankstown Hospital, Faculty of Medicine and Health, UNSW Sydney, Sydney, NSW, Australia; ^7^Department of Neurodegenerative Disease and Queen Square Brain Bank, Queen Square Institute of Neurology, University College London, London, United Kingdom; ^8^School of Medical, Indigenous and Health Sciences, University of Wollongong, Wollongong, NSW, Australia

**Keywords:** human brain banking, brain health, precision neurology, neurogenetics, neuropathology, biomarkers

Maintaining brain health and promoting precision neurology are endeavors with different perspectives but sharing the same goal of combatting brain disorders. Precision neurology is an off-shoot of precision medicine, an initiative announced by President Obama in 2015 to develop prevention and treatment strategies that take individual variability into account (1). Advances in neurogenetics, neuroimaging, neuropathology and other areas are now positioning neurology as a new frontier of precision medicine. As just one example, precision neurology is now being trialed to differentiate AD, Lewy body disease and mild cognitive impairment to support more precise diagnosis and targeted therapies by developing workflows that combine surveys on variables such as health behaviors with: (i) neurologic and neuropsychological assessments; (ii) neuroimaging biomarkers (MRI, amyloid PET) and (iii) whole genome sequencing (2).

Precision neurology focuses on the heterogeneity of disease phenotypes. The World Health Organization considers the complementary field of brain health from the perspective of optimizing lifelong brain functioning across cognitive, sensory, social-emotional, behavioral and motor domains. Optimal brain health is defined by the American Heart Association/American Stroke Association as the ability to function adaptively in the environment, reflected in competencies across the domains of thinking, moving and feeling (3). Maintaining optimal brain health across the lifespan is central to achieving a sustainable socioeconomic future for humankind.

Neurological diseases are a leading cause of all-age mortality and disability worldwide, second only to cardiovascular disease (4), with over one in four people developing brain diseases such as dementia or stroke during their lifetime (5). Preventing overt brain diseases affecting normal brain function is fundamental to maintaining brain health (4).

For example, in this topic, Hu et al. show multiple brain domains (e.g. perception, mood, attention, pain) can be affected in movement disorders, highlighting the need to consider the complex relationships between brain health and precision neurology in clinical practice, with individual variations across experiential domains potentially influencing both symptoms and treatment outcomes.

As this topic demonstrates, other studies are consolidating foundations for preventive and therapeutic advances in precision neurology, including genome-wide association studies (GWAS) and genome sequencing (gene panel, whole-exome or whole-genome sequencing) that open the way for incorporating genetic variants into the diagnosis and prognosis of both rare and common neurological diseases and into clinical trials of therapeutic interventions based on the patient's genetic variants. The excellent treatment outcome by Wang et al. for a patient with levodopa-resistant dopa-responsive dystonia with an atypical mutation illustrates the potential value of applying precision medicine in neurology—precision neurology.

Fortunately, while treatment resistance is common in neurological conditions, there is often a prodromal period when interventions can be implemented to prevent or delay disease, if markers of early changes can be identified. Analysis combining PET imaging for Aβ aggregates and CSF biomarker data from the Alzheimer's Disease Neuroimaging Initiative (ADNI) by Xiang et al. suggests deficiencies in confrontation naming and semantic fluency reflect abnormal brain Aβ deposition, which may serve as a clinical red flag for brain health maintenance. The potential for precise early intervention is increasing with advances in monitoring Aβ and other biomarkers in biofluids at the pre-symptomatic stage (6).

Other organs contribute to brain health (7–12) and neurological diseases can have systemic sequelae e.g., cardiovascular, immune, gastrointestinal or kidney effects (9–14). Hu et al. report on systematic problems, notably gastrointestinal, cardiovascular, urinary and sexual abnormalities, that can co-exist with movement disorders. The prevalence of comorbidity with brain disorders in the elderly underlines the value of both maintaining general wellbeing for brain health and considering causal directionality of comorbidities.

Despite advances in neuroimaging and other technologies, neuropathology remains essential for correlating observations in living patients with neuropathological features essential for definitive diagnosis and subtype delineation in many neurological conditions, notable examples including neurodegenerative diseases and chronic traumatic encephalopathy (15, 16).

If precision neurology and brain health are viewed as two sides of a coin, human brain banking is the edge that connects the two sides. Human brain banks are now being seen as core research facilities that support both clinical and fundamental researchers (16–18). Building a strong human brain bank to achieve the goals of precision neurology and brain health optimization will be assisted by prospective longitudinal observation of living donors with or without brain disease. This also provides opportunities for brain health promotion and management.

Maintaining brain health and consenting to become a brain donor are voluntary choices relying strongly on the willingness of participants. Complementing this, optimization of brain health requires correlating postmortem brain data with individual variations interrogated by precision neurology, studies resting largely on contributions from clinical and research professionals and encompassing individual behaviors, lifestyle choices, environmental exposures, clinicopathological and genomic factors and biomarker analyses.

Senesi et al. in this topic compare levels of CSF biomarkers for Creutzfeld-Jakob disease (CJD) in specimens from cases confirmed post-mortem as CJD or non-CJD, to determine the optimal cutpoints of an automated immunoassay for pre-mortem CJD diagnosis. This work illustrates the importance of definitive post-mortem brain assessment in developing clinical applications of precision neurology.

Human brain banking enables cataloging and study of distinctive characteristics of individual brains, facilitating development of novel technologies for understanding the brain in health and disease. The collection of high-quality brain specimens supported by “gold-standard” postmortem diagnostic data and rich clinical and pre-clinical information that includes participant health monitoring, genomics information and neuroimaging and other biomarker studies will form the foundations upon which successful implementation of precision neurology and brain health optimization across the lifespan will ultimately depend.

This topic aims to bring together researchers across these fields and raise awareness of the importance of human brain banking in precision neurology brain health optimization. It draws attention to the need for promoting and monitoring brain health—and individual factors which affect it—in partnership with living brain donors. This implicitly requires strengthening national and international policies to increase investment in brain banking so the aims of precision neurology and brain health optimization throughout the lifespan can be achieved throughout the world.

## Author contributions

YH: Conceptualization, Funding acquisition, Writing—original draft, Writing—review & editing. Q-XL: Validation, Conceptualization, Writing—review & editing. L-XC: Writing—original draft, Project administration. GW: Writing—review & editing, Conceptualization, Validation. DC: Writing—review & editing, Validation. CB: Funding acquisition, Writing—review & editing, Validation. AM: Validation, Writing—review & editing, Conceptualization.
